# The effect of estrogen on brown adipose tissue activity in male rats

**DOI:** 10.1186/s13104-022-05910-x

**Published:** 2022-02-08

**Authors:** W. Sievers, C. Kettle, R. A. Green, L. Van Schaik, M. W. Hale, H. R. Irving, D. R. Whelan, J. A. Rathner

**Affiliations:** 1 Department of Pharmacy and Biomedical Sciences, La Trobe University, Bendigo, VIC Australia; 2 Department of Psychology and Counselling, La Trobe University, Bundoora, VIC Australia; 3 School of Biomedical Sciences, Faculty of Medicine, Dentistry and Health Sciences, University of Melbourne, Parkville, VIC Australia; 4 Department of Rural Health, Faculty of Medicine, Dentistry and Health Sciences, University of Melbourne, Shepparton, VIC Australia

**Keywords:** Rodents, Thermogenesis, Thermoregulation, Transwomen

## Abstract

**Objective:**

Centrally administered estrogen can increase sympathetic nerve activity to brown adipose tissue, resulting in thermogenesis. The central thermogenic effects of estrogen have not been investigated in males. Therefore, this study sought to investigate the effects of peripherally and centrally administered estrogen on thermogenesis, heart rate and mean arterial pressure in male rats. Thermogenesis was assessed by monitoring brown adipose tissue temperature.

**Results:**

Peripherally administered estrogen elicited no significant effect on brown adipose tissue temperature, heart rate or mean arterial pressure. Centrally administered estrogen elicited a coincident increase in both brown adipose tissue and core temperature. Centrally administered estrogen also resulted in a decrease in mean arterial pressure but had no effect on heart rate. With the present data it is not possible to elucidate whether changes in temperature were the result of thermogenic or thermoregulatory mechanisms.

**Supplementary Information:**

The online version contains supplementary material available at 10.1186/s13104-022-05910-x.

## Introduction

Decreased brown adipose tissue (BAT) activity has been implicated in post-menopausal body weight gain. Ovariectomy is an experimentally induced animal model for menopause [9], where rats gain significant weight. Importantly, peripheral administration of exogenous estrogen is sufficient for normal bodyweight to be restored [8, 13, 19]. Estrogen also has potential for increasing BAT sympathetic nerve discharge (SND) and BAT thermogenesis in females [2, 11, 17]. Several lines of evidence suggest that the presence of estrogen receptor alpha (Esr1) within the ventromedial nucleus of the hypothalamus (VMH) is implicated in the regulation of BAT SND. Adenovirus mediated knockout of Esr1 receptors in the VMH results in weight gain [12]. Further, intracerebroventricular (ICV) administration of estrogen can increase BAT SND in ovariectomised rats. The same injections also increased the protein products of immediate early gene markers of activity (e.g. cFos protein) in VMH neurons [9].

Existing literature on male responses to estrogen is limited and studies that do include males give equivocal results [16]. For example, a deficiency of the G-protein coupled estrogen receptor (Gper) or Esr1 in male mice induces insulin resistance and obesity [4, 15], however it was not established whether an increase in Gper or Esr1 signaling would have the opposite effect. Peripheral administration of estrogen increases physical activity in male rats, but to a lesser degree than females [10]. Estrogen is the primary female sex hormone, however, it is important to study its action in males. One example of clinical translation in this area relates to transwomen (individuals assigned as male at birth, but who now identify with feminine gender expression). Transwomen undergoing feminizing hormone therapy (antiandrogen and exogenous estrogen) have been reported to suffer an increase in body mass index (BMI) and adiposity [5]. Hormone therapy among transwomen has also been correlated with increased cardiovascular morbidity [1, 3]. It remains unclear whether these side effects are mediated by antiandrogen or estrogen mechanisms, or a combination thereof.

Both peripheral administration (intraperitoneal (IP), intravenous (IV) or subcutaneous (SC) injection) and central administration (intracerebroventricular (ICV) injection) of estrogen successfully elicit an increase in BAT thermogenesis in female rats [8, 9, 12, 19]. There is limited literature assessing the metabolic outcomes of these interventions in male rats. Therefore, the aim of these experiments is to investigate whether peripheral or central administration of estrogen acutely stimulates BAT thermogenesis in male rats. A secondary aim is to assess if estrogen stimulates any adverse side effects on the cardiovascular system (changes to heart rate or MAP).

## Main text

### Materials and methods

To test whether estrogen influences iBAT thermogenesis we employed a parallel study design. Estrogen or vehicle was administered either peripherally (via IP injection, vehicle = sesame oil), or centrally (via ICV injection, vehicle = saline + 10% DMSO). These protocols have been used previously [18] and are described briefly here.

#### Animals

Male Sprague-Dawley rats (Monash Animal Services) (350–400 g) were randomly allocated to one of the four treatment groups (IP control, IP estrogen, ICV control, ICV estrogen), by use of a random number generator. Confounders were controlled by randomizing the order of treatments. Two investigators were involved in all experimental steps; one primary (blinded) and one assistant (unblinded). The assistant investigator was responsible for allocation of rats and preparing the treatment/control to be administered during the experiment, while maintaining blinding of the primary investigator. The primary investigator was responsible for conduction of the experiment and the outcome assessment. The primary investigator was unblinded for data analysis. One rat was considered one experimental unit. A power analysis was performed a priori using G-Power, based on data extracted from Martinez de Morentin et al. [9]. Assuming an increase in iBAT temperature of 0.7 °C, with a standard deviation of 0.56 °C, following 10 μg/kg administration of estrogen via IP injection. This power analysis indicated that eight animals per treatment group were required. All experiments performed in this study were approved by the La Trobe University Animal Ethics Committee (Ethics approval number: AEC16-02). Anesthetic depth was routinely monitored in compliance with animal ethics standards. All efforts were made to limit the number of animals used and their suffering. Criteria for including or excluding animals or data were not established a priori. Data from experiments that were terminated early, or suffered prolonged delay (> 7 h) between anesthetic induction and administration of intervention due to surgical complications, were not included in analyses.

#### Experimental procedure

Rats in the IP administration group were injected with estrogen (5–10 µg/kg/0.3 ml, Sigma-Aldrich) [9] or sesame oil (vehicle only, 0.3 ml, Sigma-Aldrich). Rats in the ICV administration group were injected with estrogen (20 ng/200 nl) or saline + 10% dimethyl sulfoxide (DMSO) (vehicle only, 200 nl, Sigma-Aldrich). Four hours post administration of treatment, the animals were euthanized by transcardial perfusion under urethane-anesthesia, and the brains were prepared for cFos immunohistochemical analysis. Tissue processing and immunohistochemistry was performed as described by Lawther et al. [7]. Complete details of the cFos immunohistochemistry method are in Additional file 1.

#### Statistical analysis

Statistical analysis of all outcomes (temperature, heart rate, MAP) was performed using GraphPad PRISM 9. Averages of iBAT temperature, core temperature, heart rate, and MAP were calculated over 1-min at 5-min intervals, until 210-min post intervention. These data were then expressed as a change from baseline. Statistical significance was tested using a two-way mixed-model ANOVA. Separate ANOVAs were conducted for each administration method (IP-control versus IP-estrogen, and ICV-control versus ICV-estrogen). ANOVAs are parametric tests, that assume normally distributed data. Normality tests were performed, which indicated several variables were not normally distributed, likely because of small values for n. ANOVAs were still used because the central limit theorem states that given sufficient samples, sample distribution will be normal, regardless of the underlying population distribution [6].

### Results

#### Central vs peripheral effects of estrogen on iBAT and core temperature

Core and iBAT temperature were both monitored via thermocouples. Heart rate and blood pressure were measured using an indwelling catheter inserted in the carotid artery. A stable baseline was ensured for a minimum of 30 min prior to injection. No differences in temperature baseline conditions occurred although some differences in cardiovascular baseline conditions were evident between treatment groups (Table 1).

**Table 1:** Average baseline of physiological metrics, 15-min prior to administration of intervention, ± represents SD

Route of administration	Treatment	Core temperature (°C)	iBAT temperature (°C)	Heart rate (bpm)	MAP (mmHg)
IP	Control	36.42 ± 0.20	34.71 ± 0.62	387 ± 27	133.00 ± 5.92
IP	Estrogen	36.50 ± 0.30	34.90 ± 1.23	426 ± 49	145.77 ± 9.14**
ICV	Control	36.60 ± 0.27	35.65 ± 0.72	394 ± 33	120.90 ± 15.24
ICV	Estrogen	36.37 ± 0.46	34.82 ± 0.69	427 ± 17*	160.27 ± 7.37***

Statistical testing indicates some difference between estrogen and control conditions with the same routes of administration (IP or ICV) in cardiovascular baseline conditions prior to intervention. p-value calculated by unpaired, two-tailed t-test, n = 6 for IP-control; n = 7 for IP-estrogen and ICV-control; n = 8 for ICV-estrogen. *Indicates p < 0.05, **indicates p < 0.01, ***indicates p < 0.001. One rat was excluded from the IP-control group due to a procedural error. Two rats were excluded from the ICV-estrogen group due to prolonged (< 7 h) surgical complications experienced

**Fig. 1:**
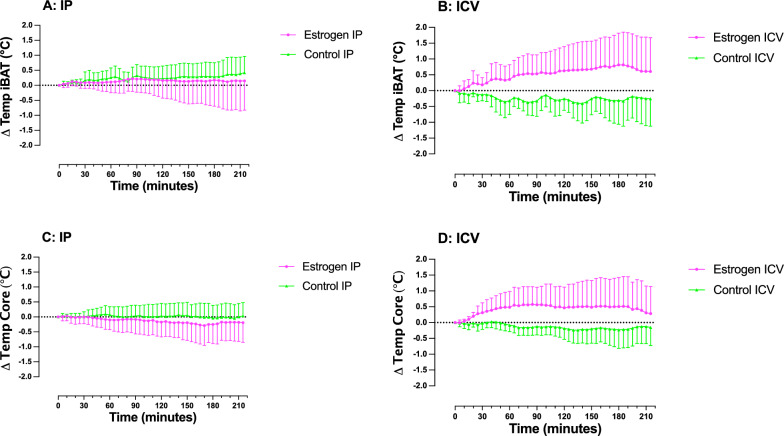
Changes in temperature (Δ Temperature °C) of interscapular brown adipose tissue (iBAT) and core, in male rats following injection (time = zero) of estrogen or vehicle. Temperature of iBAT following **A** IP injection or **B** ICV injection. Core temperature following **C** IP injection or **D** ICV injection. Error bars represent SD. Statistical significance was tested using a two-way mixed model ANOVA. Separate ANOVAs were conducted for each route of administration (IP-control versus IP-estrogen, and ICV-control versus ICV-estrogen). n = 6 for IP-control; n = 7 for IP-estrogen and ICV-control; n = 8 for ICV-estrogen. One rat was excluded from the IP-control group due to a procedural error. Two rats were excluded from the ICV-estrogen group due to prolonged (< 7 h) surgical complications experienced

Following IP administration of estrogen no interaction effect (time × treatment) was observed for iBAT temperature (F_(43, 473)_ = 0.25, p > 0.99, Fig. 1A), or core temperature (F_(43, 473)_ = 0.50, p > 0.99, Fig. 1C). Šidák’s multiple comparisons test revealed no significant difference in iBAT temperature at any time point (at 150 min: mean difference = 0.14 °C, p > 0.99, 95% C.I. [− 1.37, 1.67]), or core temperature (at 150 min: mean difference = 0.24 °C, p > 0.99, 95% C.I. [− 1.02, 1.49]).

A significant interaction effect on iBAT temperature was observed following ICV administration of estrogen (F_(43, 559)_ = 2.25, p < 0.001, Fig. 1B). A corresponding rise in core temperature was observed following ICV injection of estrogen (F_(43, 559)_ = 1.60, p = 0.01, Fig. 1D). Despite the overall significant difference in response, between ICV control and ICV estrogen treated rats, Šidák’s multiple comparisons test showed no significant difference at any given time point. iBAT temperature (at 150 min: mean difference = − 0.93 °C, p = 0.71, 95% C.I. [− 2.52, 0.66]), core temperature (at 150 min: mean difference = − 0.68 °C, p = 0.96, 95% C.I. [− 2.18, 0.82]). The varied response to estrogen administered via ICV injection is illustrated by the individual traces for each rat (Additional file 2: Figure S1).

#### Central estrogen influences MAP, but not heart rate

**Fig. 2:**
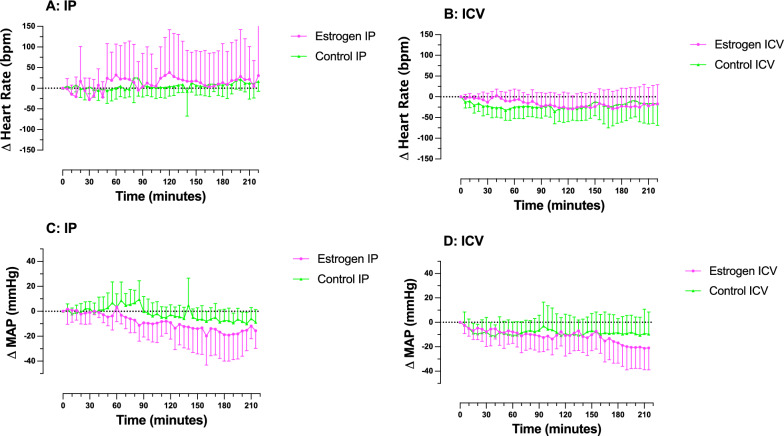
Changes in heart rate (Δ Heart Rate) and mean arterial pressure (Δ MAP), in male rats following injection (time = zero) of estrogen or vehicle. Change in heart rate following **A** IP injection or **B** ICV injection. Change in MAP following **C** IP injection or **D** ICV injection. Error bars represent SD. Statistical significance was tested using a two-way mixed model ANOVA. Separate ANOVAs were conducted for each route of administration (IP-control versus IP-estrogen, and ICV-control versus ICV-estrogen). n = 6 for IP-control; n = 7 for IP-estrogen and ICV-control; n = 8 for ICV-estrogen. One rat was excluded from the IP-control group due to a procedural error. Two rats were excluded from the ICV-estrogen group due to prolonged (< 7 h) surgical complications experienced

Following administration of estrogen by IP injection, no significant effect was observed on heart rate (F_(44, 484)_ = 0.66, p > 0.95, Fig. 2A) or MAP (F_(43, 473)_ = 0.82, p > 0.78, Fig. 2C). Šidák’s multiple comparisons test found no significant difference in heart rate (at 150 min: mean difference = − 11 bpm, p > 0.99, 95% C.I. [− 162, 139]), or MAP (at 150 min: mean difference = 7 mmHg, p > 0.99, 95% C.I. [− 33.25, 47.88]).

Following ICV administration of estrogen no significant effect was observed on heart rate (F_(44, 572)_ = 1.20, p > 0.18, Fig. 2B), however a decrease MAP (F_(43, 559)_ = 1.43, p > 0.041, Fig. 2D) was identified. Despite the overall significant difference in MAP, between ICV control and ICV estrogen treated rats, Šidák’s multiple comparisons test found that there was no significant difference in heart rate at any given time point (at 150 min: mean difference = 12 bpm, p > 0.99, 95% C.I. [− 59, 83]), or MAP (at 150 min: mean difference = 3.18 mmHg, p > 0.99, 95% C.I. [− 26.44, 32.80]). Additional file 3: Figure S2 shows individual heart rate and MAP traces for each rat.

#### cFos expression

Following temperature, heart rate and blood pressure monitoring, animals were transcardially perfused, and brains extracted. cFos immunoreactivity (cFos-IR) was assessed within specific diencephalic nuclei but no significant changes were detected (Additional file 4: Figure S3).

#### Comparison of control data

Control temperature, heart rate and MAP data were compared to data from Van Schaik et al. [18]. No differences were identified between the two studies for any metrics (Additional file 1: Table S1).

### Discussion

Outcomes observed in this study indicate that estrogen had no significant effect on iBAT temperature or core temperature when administered peripherally. Centrally administered estrogen significantly increases iBAT and core temperature in male rats. Peripheral administration of estrogen did not effect heart rate or MAP. Central administration of estrogen elicited a decrease in MAP, but not heart rate.

The findings from these experiments reiterate findings from a previous systematic review, that thermogenic effects of estrogen in male rodents are diminished or altogether absent relative to females [16]. One possible explanation for the diminished thermogenic response is that the presence of endogenous testosterone modulates receptor expression. Brown adipocytes (primary cell line) cultured in the presence of testosterone express relatively more α2A-adrenoceptors, and fewer β3-adrenoceptors [14]. As activating α2A-adrenoceptors inhibits BAT thermogenesis, and activating β3-adrenoceptors stimulates BAT thermogenesis, biasing the expression ratio towards α2A-adrenoceptors desensitises the tissue to noradrenaline [14]. Therefore, incubating brown adipocytes in testosterone results in cells that are more resistant to sympathetic activation by noradrenaline [14].

Given the differences that have been identified in the baseline conditions of our cardiovascular data, control data were compared to previously published work that used similar protocols [18]. Different vehicles were used as Van Schaik et al. [18] used saline for both IV and ICV injection, while this study used sesame oil by IP injection and 10% DMSO for ICV injection. No significant differences were found in the mean changes in the control temperature, heart rate or MAP between the two studies (Additional file 1: Table S1). Given the comparability of this data being from the same laboratory, using the same protocol, observed over the same time frame; we can offer no explanation for the differences in cardiovascular metrics between groups at baseline in this study. However, findings related to changes in cardiovascular metrics may result from these differences in baseline conditions. But, differences in anesthetic depth can be ruled out as regular monitoring was performed. Animals were also in good health as evidenced by absence of fever.

Although we observed no significant increase or decrease in thermogenesis following peripheral administration of estrogen, we did observe an increase in iBAT temperature following central administration. Estrogen can increase sympathetic nerve activity in female rats, leading to an increase in iBAT temperature [9]. In this study, a significant change in iBAT temperature occurred in male rats, however this was accompanied by a coincident change in core temperature. These coincident observations raise the possibility that increases in iBAT temperature are secondary to the core temperature increase, and not the other way around. Although it is possible to separate evoked thermogenic response from core temperature changes [18] by assessing cFos expression in known thermogenic central neural circuits. Unfortunately, we were unable to assess activation of specific nuclei by cFos expression due to tissue quality problems leaving insufficient samples for statistical analysis (n = 3). This under-powered data does not indicate any substantive difference in cFos expression between any nucleus or treatment group.

In both routes of administration (peripheral and central), responses for estrogen treated animals were more highly varied than those of the control animals. A highly inconsistent response to estrogen observed in this study highlights a need for further research. In particular, research that addresses concerns surrounding transwomen undergoing hormone therapy. These individuals have been reported to have increased BMI and adiposity [5], and increased cardiovascular morbidity [1, 3]. Further investigation is required into the effects of estrogen in males, and the mechanisms through which it is eliciting these effects. If any central pathways are involved, it may facilitate development of new hormone therapies or dosing regimens to produce the desired secondary sex characteristics, without unwanted side effects for transwomen.

## Conclusion

Peripheral administration of estrogen elicited no significant effects on iBAT temperature, core temperature, heart rate, or MAP in male rats. However, an increase in iBAT and core temperature was observed in response to estrogen administered via ICV injection. These findings reiterate those of a recent systematic review [16]; males have a diminished (or absent) thermogenic response to estrogen compared to females. The inconsistent nature of the responses to estrogen highlights the need for further research into the effects of estrogen in males. This area of research is potentially important for negating metabolic and cardiovascular side effects in transwomen undergoing hormone therapy.

## Limitations

Differences in MAP between groups, at baseline, is one limitation of the present study. This series of experiments were performed in parallel with another, published set of experiments [18], following identical procedures where no significant differences were observed among baseline conditions. Further, control data obtained from experiments, during post-intervention observational period was comparable between the two studies (Additional file 1: Table S1). As such, we are unable to suggest any systematic reason why this data has baseline differences.

Core temperature increased, as well as iBAT temperature, so it is not possible to rule out thermoregulation as a cause. cFos immunoreactivity (cFos-IR) would have made it possible to visualize which nuclei had been activated or inhibited, following a given intervention. Obtaining evidence about which nuclei were implicated in changes in iBAT and core temperature observed might have allowed inference of some underlying mechanisms. Without sufficient cFos expression data it is not possible to say whether thermogenic or thermoregulatory mechanisms were the cause of the increase in temperature observed. Molecular analysis of uncoupling protein 1 (UCP1) or downstream signaling of adrenergic receptors may also have helped determine the etiology of BAT temperature changes. Chronic experiments would provide data relating to changes in body weight and adiposity following estrogen treatment.

## Supplementary Information

**Additional file 1.** **Table S1:** Mean changes in core temperature, iBAT temperature, heart rate and mean arterial pressure for the present study (Sievers et al.) and Van Schaik et al. (2). A t-test was used to assess statistical difference between means. Mean change in temperatures are represented in the centre column. P-values are reported in the right-most column. n = 6–8.**Table S2:** Animal research: Reporting of in vivo experiments (ARRIVE) checklist.

**Additional file 2: Figure S1.** Individual changes in temperature (Δ Temperature °C) of interscapular brown adipose tissue (iBAT) and core, in male rats following injection (time = zero) of estrogen or vehicle. Temperature of iBAT following A IP injection or B ICV injection. Core temperature following C IP injection or D ICV injection. n = 6 for IPcontrol; n = 7 for IP-estrogen and ICV-control; n = 8 for ICV-estrogen. One rat was excluded from the IP-control group due to a procedural error. Two rats were excluded from the ICV-estrogen group due to prolonged (< 7 h) surgical complications experienced.

**Additional file 3: Figure S2.** Individual changes in heart rate (Δ Heart Rate) and mean arterial pressure (Δ MAP), in male rats following injection (time = zero) of estrogen or vehicle. Heart rate following A IP injection or B ICV injection. Mean arterial pressure following C IP injection or D ICV injection. n = 6 for IP-control; n = 7 for IP-estrogen and ICV-control; n = 8 for ICV-estrogen. One rat was excluded from the IP-control group due to a procedural error. Two rats were excluded from the ICV-estrogen group due to prolonged (< 7 h) surgical complications experienced.

**Additional file 4: Figure S3.** Number of cFos immunoreactive (cFos-ir) cells within thalamic and hypothalamic nuclei. n = 3 for all treatment groups. One rat was considered one experimental unit. VMH = ventromedial nucleus of the hypothalamus; Arc = arcuate nucleus of the hypothalamus; LH = lateral nucleus of the hypothalamus; PVN = Paraventricular nucleus of the hypothalamus; PVT = paraventricular nucleus of the thalamus; CM = centromedian nucleus of the thalamus; DMH = dorsomedial nucleus of the hypothalamus.

## Data Availability

The datasets used and/or analyzed during the current study are available from the corresponding author on reasonable request.

## Abbreviations

ANOVA: Analysis of variance
BAT: Brown adipose tissue
iBAT: Interscapular brown adipose tissue
BMI: Body mass index
cFos-IR: CFos immunoreactivity
DMSO: Dimethyl sulfoxide
Esr1: Estrogen receptor 1
Gper: G-protein coupled estrogen receptor
ICV: Intracerebroventricular
IP: Intraperitoneal
IV: Intravascular
MAP: Mean arterial pressure
SC: Subcutaneous
SD: Standard deviation
SND: Sympathetic nerve discharge
UCP1: Uncoupling protein 1
VMH: Ventromedial hypothalamus
