# Supplementary material for: The effect of estrogen on brown adipose tissue activity in male rats
Source: BMC Res Notes. 2022 Feb 8;15:28. doi: 10.1186/s13104-022-05910-x (PMC8822813; doi:10.1186/s13104-022-05910-x)
Supplement: Supplementary file 1 — Additional file 1. Table S1: Mean changes in core temperature, iBAT temperature, heart rate and mean arterial pressure for the present study (Sievers et al.) and Van Schaik et al. (2). A t-test was used to assess statistical difference between means. Mean change in temperatures are represented in the centre column. P-values are reported in the right-most column. n = 6–8.Table S2: Animal research: Reporting of in vivo experiments (ARRIVE) checklist. [file 13104_2022_5910_MOESM1_ESM.docx]

**Additional file 1**

**Individual Data**

Individual traces are presented for temperature, heart rate and mean arterial pressure. This is in order to assist the reader in visualizing the spread of our data.

**[insert figure S1 here]**

**Figure S1:** Temperature changes in interscapular brown adipose tissue (iBAT) and core for individual male rats following injection of estrogen or vehicle. Temperature of iBAT following (A) IP injection or (B) ICV injection. Core temperature following (C) IP injection or (D) ICV injection.

**[insert figure S2 here]**

**Figure S2:** Changes in heart rate and mean arterial pressure (MAP) for individual male rats following injection of estrogen or vehicle. Heart rate following (A) IP injection or (B) ICV injection. MAP following (C) IP injection or (D) ICV injection.

**c-Fos Methods**

**Perfusion and Tissue Processing**

Upon completion of the experimental period animals were transcardially perfused using 10% neutral buffered (pH 7.4) formalin 4% formaldehyde solution. Extracted brains were post-fixed in the same neutral-buffered formalin solution for 12 hours. Tissue was cryopreserved for 24 hours in 30% sucrose in 0.1M phosphate-buffered saline (PBS), prior to processing and immunohistochemistry. Tissue processing and immunohistochemistry was performed as described by Lawther et al. (1).

**Antibodies for c-Fos immunohistochemistry**

Immunohistochemistry was performed on 30 μm sections of brain tissue from the hypothalamic region. An avidin-biotin-horseradish peroxidase complex (ABC) kit, with rabbit anti-cFos-polyclonal antibody (1:3000; catalogue no. ABE457;EMD Millipore Corp, USA), a biotinylated anti-rabbit IgG antibody (1:200; Vector Laboratories, Burlingame, CA, USA), ABC (1:200; Vectastain Elite ABC Kit; Vector Laboratories, Burlingame, CA, USA), and Vector SG (Peroxidase substrate kit; catalogue no. SK-4700; Vectastain Elite ABC Kit; Vector Laboratories, Burlingame, CA, USA) to produce blue puncta of c-Fos-positive cells.

**Cell counts – VMH, Arc, LH, PVN, PVT, CM, DMH (dorsal)**

Sections of brain tissue sections from the ICV administration group, between the rostrocaudal levels of -2.12mm and -3.60mm from bregma, containing the ventromedial hypothalamus (VMH), arcuate nucleus of the hypothalamus (Arc), lateral hypothalamus (LH), paraventricular nucleus of the hypothalamus (PVN), paraventricular nucleus of the thalamus (PVT), centromedial thalamus (CM), dorsal region of the dorsomedial hypothalamus (DMH) were selected for analysis (the DMH was only present between -2.56mm and 3.60mm from bregma). Round-shaped nuclei (circularity between 0.8-1.0) with dark blue to black colour were counted as c-Fos immunoreactive (cFos-IR). Microscopy, automated c-Fos immunoreactive cell counting, and reliability testing was performed as per Van Schaik et al. (2).

**Statistical Analysis – cFos-IR**

Separate ANOVAs were conducted for each administration method (IP-control vs IP-estrogen, and ICV-control vs ICV-estrogen). Brain tissue cFos immunoreactivity counts were averaged for each nucleus of interest, and analysed by one-way ANOVA with all nuclei.

**c-Fos Results**

**Estrogen does not influence c-Fos expression**

In order to investigate whether thermogenic neurocircuitry was being activated in the hypothalamic region of the brain, cFos immunohistochemistry was performed. Dark field microscopy was used to more accurately identify the neuroanatomy, for precise tracing of nuclei. Bright field microscopy and automated particle counting was used to quantify the cFos-immunoreactive cells within nuclei of interest. The reliability of the automated cell counting protocol was tested against a blinded assessor and calculating the intraclass correlation coefficient (ICC) revealed very good reliability (ICC_3,1_ = 0.99). No effect on cFos-IR was observed (Figure S3), indicating that thermogenic circuitry might not have been activated. However, the n-value is not high enough to draw any conclusions from this data.

**[insert figure S3 here]**

**Figure S3:** Average number of cFos imunoreactive cells per section (# cFos-ir cells) for hypothalamic and thalamic nuclei following administration of estrogen via ICV injection. (A) ventromedial hypothalamus (VMH); (B) arcuate nucleus of the hypothalamus (Arc); (C) lateral hypothalamus (LH); (D) paraventricular nucleus of the hypothalamus (PVN); (E) paraventricular nucleus of the thalamus (PVT); (F) centromedial thalamus (CM); (G) dorsal region of the dorsomedial hypothalamus (DMH). Graphs represent mean number of cFos-IR cells as an average for each nucleus. Each point on the graph represents the average for an individual rat. Error bars represent SD, n = 3 per treatment. Statistical significance was assessed by one-way ANOVA for all nuclei.

**Control data for core temperature, iBAT temperature, heart rate and mean arterial pressure compared to Van Schaik et al.**

Mean differences and p-values have been tabulated in order to compare control data from the present study with that from Van Schaik et al. (2).

**Table S1: Comparison of Control Data to Van Schaik et al.**

|  | Δ mean (temperature = °C ± SD) (heart rate = bpm) (mean arterial pressure = mmHg) | p-value |
| --- | --- | --- |
| Core temperature (peripheral administration) | Sievers et al. (sesame oil) 0.028 ± 0.29 | 0.94 |
|  | Van Schaik et al. (saline) 0.018 ± 0.24 |  |
| Core temperature (ICV administration) | Sievers et al. (10% DMSO) -0.11 ± 0.27 | 0.94 |
|  | Van Schaik et al. (saline) -0.097 ± 0.40 |  |
| iBAT temperature (peripheral administration) | Sievers et al. (sesame oil) 0.20 ± 0.31 | 0.26 |
|  | Van Schaik et al. (saline) 0.0092 ± 0.34 |  |
| iBAT temperature (ICV administration) | Sievers et al. (10% DMSO) -0.24 ± 0.39 | 0.26 |
|  | Van Schaik et al. (saline) -0.03 ± 0.39 |  |
| Heart rate (peripheral administration) | Sievers et al. (sesame oil) 3.18 ± 25.81 | 0.25 |
|  | Van Schaik et al. (saline) -14.18 ± 40.24 |  |
| Heart rate (ICV administration) | Sievers et al. (10% DMSO) -23.05 ± 25.44 | 0.98 |
|  | Van Schaik et al. (saline) -22.67 ± 43.96 |  |
| Mean arterial pressure (peripheral administration) | Sievers et al. (sesame oil) 0.28 ± 10.43 | 0.46 |
|  | Van Schaik et al. (saline) -2.93 ± 12.97 |  |
| Mean arterial pressure (ICV administration) | Sievers et al. (10% DMSO) -7.99 ± 11.84 | 0.99 |
|  | Van Schaik et al. (saline) -8.05 ± 12.08 |  |

**Table S1:** Mean changes in core temperature, iBAT temperature, heart rate and mean arterial pressure for the present study (Sievers et al.) and Van Schaik et al. (2). A t-test was used to assess statistical difference between means. Mean change in temperatures are represented in the centre column. P-values are reported in the right-most column. n = 6-8.

**References**

1. **Lawther AJ, Flavell A, Ma S, Kent S, Lowry CA, Gundlach AL, and Hale MW.** Involvement of Serotonergic and Relaxin-3 Neuropeptide Systems in the Expression of Anxiety-like Behavior. *Neuroscience* 390: 88-103, 2018.

2. **Van Schaik L, Kettle C, Green R, Sievers W, Hale MW, Irving HR, Whelan DR, and Rathner JA.** Stimulatory, but not anxiogenic, doses of caffeine act centrally to activate interscapular brown adipose tissue thermogenesis in anesthetized male rats. *Scientific Reports* 11: 113, 2021.
