# Supplementary figures and images for: The effect of estrogen on brown adipose tissue activity in male rats
Source: BMC Res Notes. 2022 Feb 8;15:28. doi: 10.1186/s13104-022-05910-x (PMC8822813; doi:10.1186/s13104-022-05910-x)

**A: IP**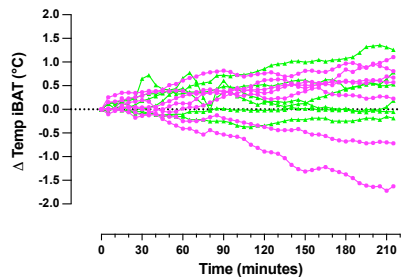**B: ICV**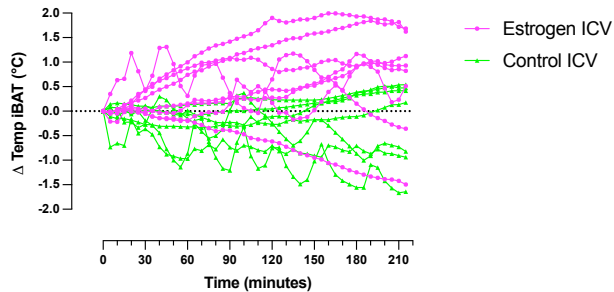**C: IP**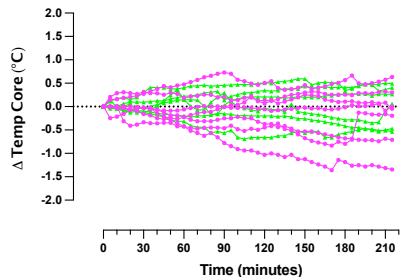**D: ICV**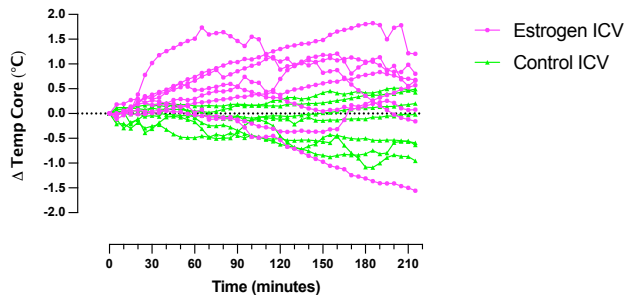

Supplement: Supplementary file 2 — Additional file 2: Figure S1. Individual changes in temperature (Δ Temperature °C) of interscapular brown adipose tissue (iBAT) and core, in male rats following injection (time = zero) of estrogen or vehicle. Temperature of iBAT following A IP injection or B ICV injection. Core temperature following C IP injection or D ICV injection. n = 6 for IPcontrol; n = 7 for IP-estrogen and ICV-control; n = 8 for ICV-estrogen. One rat was excluded from the IP-control group due to a procedural error. Two rats were excluded from the ICV-estrogen group due to prolonged (< 7 h) surgical complications experienced. [file 13104_2022_5910_MOESM2_ESM.pdf]

**A: IP**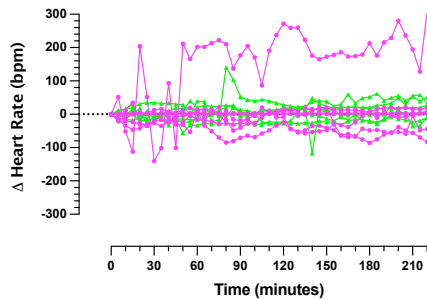**B: ICV**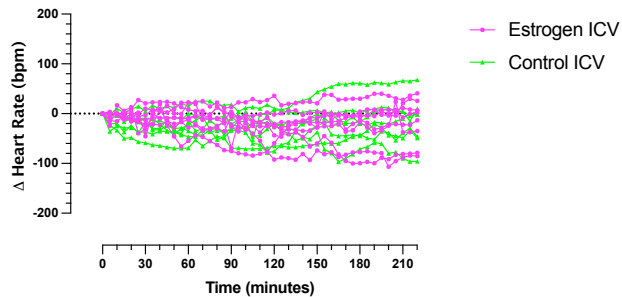**C: IP**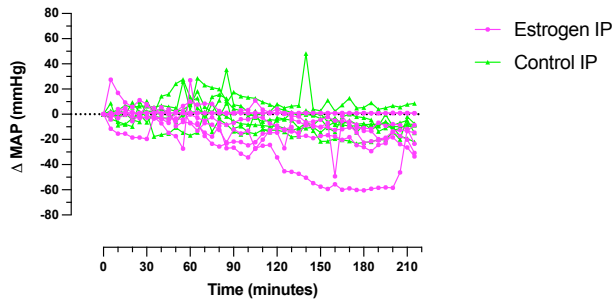**D: ICV**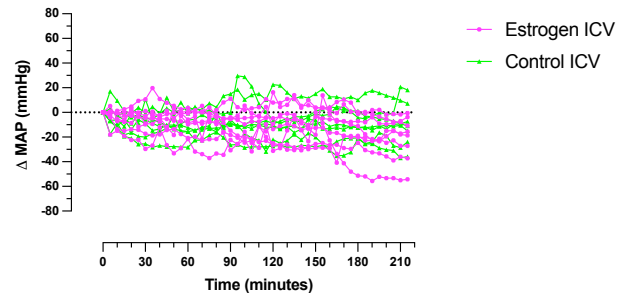

Supplement: Supplementary file 3 — Additional file 3: Figure S2. Individual changes in heart rate (Δ Heart Rate) and mean arterial pressure (Δ MAP), in male rats following injection (time = zero) of estrogen or vehicle. Heart rate following A IP injection or B ICV injection. Mean arterial pressure following C IP injection or D ICV injection. n = 6 for IP-control; n = 7 for IP-estrogen and ICV-control; n = 8 for ICV-estrogen. One rat was excluded from the IP-control group due to a procedural error. Two rats were excluded from the ICV-estrogen group due to prolonged (< 7 h) surgical complications experienced. [file 13104_2022_5910_MOESM3_ESM.pdf]

**A: VMH**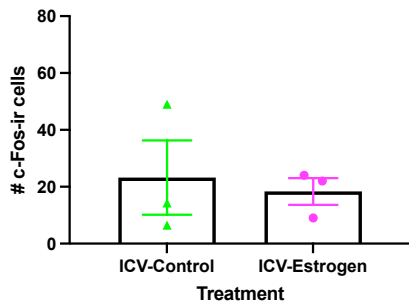**B: Arc**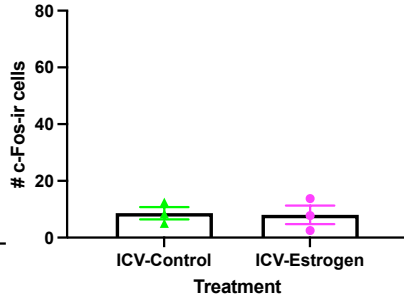**C: LH**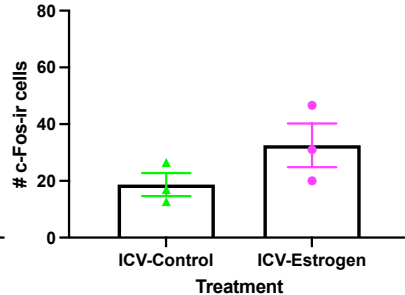**D: PVN**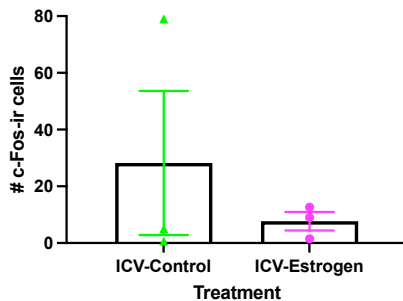**E: PVT**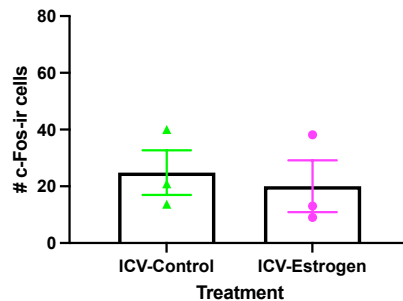**F: CM**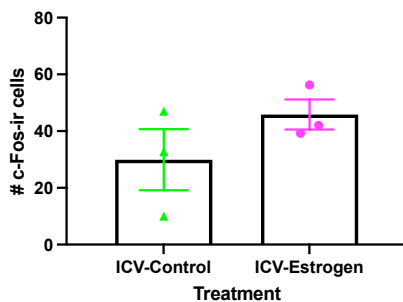**G: DMH**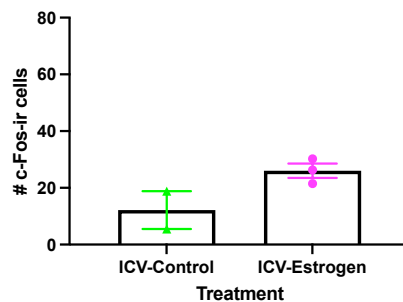

Supplement: Supplementary file 4 — Additional file 4: Figure S3. Number of cFos immunoreactive (cFos-ir) cells within thalamic and hypothalamic nuclei. n = 3 for all treatment groups. One rat was considered one experimental unit. VMH = ventromedial nucleus of the hypothalamus; Arc = arcuate nucleus of the hypothalamus; LH = lateral nucleus of the hypothalamus; PVN = Paraventricular nucleus of the hypothalamus; PVT = paraventricular nucleus of the thalamus; CM = centromedian nucleus of the thalamus; DMH = dorsomedial nucleus of the hypothalamus. [file 13104_2022_5910_MOESM4_ESM.pdf]
